# Association Between Oxidative Potential of Particulate Matter Collected by Personal Samplers and Systemic Inflammation Among Asthmatic and Non-Asthmatic Adults

**DOI:** 10.3390/antiox13121464

**Published:** 2024-11-28

**Authors:** Miguel Santibáñez, Juan José Ruiz-Cubillán, Andrea Expósito, Juan Agüero, Juan Luis García-Rivero, Beatriz Abascal, Carlos Antonio Amado, Laura Ruiz-Azcona, Marcos Lopez-Hoyos, Juan Irure, Yolanda Robles, Ana Berja, Esther Barreiro, Adriana Núñez-Robainas, José Manuel Cifrián, Ignacio Fernandez-Olmo

**Affiliations:** 1 Global Health Research Group, Faculty of Nursing, Universidad de Cantabria-Valdecilla Research Institute (IDIVAL), Avenida Valdecilla, s/n, 39008 Santander, Cantabria, Spain; laura.ruiz@unican.es; 2 Division of Pneumology, Hospital Universitario Marqués de Valdecilla, IDIVAL, 39008 Santander, Cantabria, Spain; juanjose.ruizc@scsalud.es (J.J.R.-C.); jagcal@gmail.com (J.A.); jgarcia@separ.es (J.L.G.-R.); beatriz.abascal@scsalud.es (B.A.); amadodiago.carlos@gmail.com (C.A.A.); josemanuel.cifrian@scsalud.es (J.M.C.); 3 Departamento de Ingenierías Química y Biomolecular, Universidad de Cantabria, Avenida Los Castros, s/n, 39005 Santander, Cantabria, Spain; andreaexpositomonar@gmail.com (A.E.); ignacio.fernandez@unican.es (I.F.-O.); 4 Division of Immunology, Hospital Universitario Marqués de Valdecilla, IDIVAL, 39008 Santander, Cantabria, Spain; marcos.lopez@scsalud.es (M.L.-H.); juan.irure@scsalud.es (J.I.); 5 Division of Biochemistry, Hospital Universitario Marqués de Valdecilla, IDIVAL, 39008 Santander, Cantabria, Spain; roblesfla@hotmail.es (Y.R.); anaberj@gmail.com (A.B.); 6 Pulmonology Department-Muscle Wasting and Cachexia in Chronic Respiratory Diseases and Lung Cancer, IMIM-Hospital del Mar, Department of Medicine and Life Sciences (MELIS), Universitat Pompeu Fabra, Barcelona Biomedical Research Park (PRBB), 08003 Barcelona, Spain; ebarreiro@imim.es (E.B.); adriananunez96@gmail.com (A.N.-R.); 7 Centro de Investigación en Red de Enfermedades Respiratorias (CIBERES), Instituto de Salud Carlos III (ISCIII), 08034 Barcelona, Spain

**Keywords:** particulate matter (PM), oxidative potential (OP), asthma, systemic inflammation, interleukin-6

## Abstract

With the rationale that the oxidative potential of particulate matter (PM-OP) may induce oxidative stress and inflammation, we conducted the ASTHMA-FENOP study in which 44 asthmatic patients and 37 matched controls wore a personal sampler for 24 h, allowing the collection of fine and coarse PM fractions separately, to determine PM-OP by the dithiothreitol (DTT) and ascorbic acid (AA) methods. The levels of Interleukin 6 (IL-6) and the IL-6/IL-10 ratio, as indicators of pro- and anti-inflammatory statuses, were determined by calculating the mean differences (MDs), odds ratios (ORs) and p-trends adjusted for sex, age, study level and body mass index. Positive associations for IL-6 levels in the form of adjusted MDs and ORs were obtained for all PM-OP metrics, reaching statistical significance for both OP-DTT and OP-AA in the fine fraction, with adjusted OR = 5.66; 95%CI (1.46 to 21.92) and 3.32; 95%CI (1.07 to 10.35), respectively, along with statistically significant dose–response patterns when restricting to asthma and adjusted also for clinical variables (adjusted p-trend = 0.029 and 0.01). Similar or stronger associations and dose–response patterns were found for the IL-6/IL-10 ratio. In conclusion, our findings on the effect of PM-OP on systemic inflammation support that asthma is a heterogeneous disease at the molecular level, with PM-OP potentially playing an important role.

## 1. Introduction

Among air pollutants, particulate matter (PM) has the greatest impact on human health [1,2]. In this regard, the toxicity of PM, beyond its chemical composition, seems to be related to its capacity to generate reactive oxygen species (ROS). This may alter the balance between oxidants and antioxidants in the cells in favor of the former, leading to oxidative stress [3]. Increased expression of inflammatory cytokines and other molecules, such as cellular adhesion molecules and coagulation factors, may also be involved [2].

The characterization of PM exposure in epidemiological studies is mainly based on the PM mass concentration and chemical composition from filters collected by stationary samplers. However, recent studies have suggested that the oxidative potential (OP) of PM may be a better proxy than the PM mass concentration to account for the exposure to PM in such studies [4]. PM OP is defined as the ability of inhaled components to produce ROS while simultaneously depleting antioxidants [5]. In addition, this characterization is usually not performed on an individual basis. In this sense, the use of personal PM samplers instead of stationary ones allows the collection of particles to which a volunteer has been exposed to in the last 24 h. These personal PM samplers have been used in some studies with the aim of determining the mass and chemical composition of PM [6,7] and, recently, its OP also [5,8,9,10,11].

Asthma is currently the most prevalent chronic respiratory disease worldwide, with twice as many cases as Chronic Obstructive Pulmonary Disease (COPD) [12]. There is consistent evidence on the association between air pollution and a higher incidence of asthma [13]. In relation to the clinical course of this disease, there is evidence from meta-analyses and subsequent studies on the effects of airborne PM on the use of rescue medication, visits to emergency departments, and especially, admissions for asthma exacerbations [14,15]. Recently, an intervention reducing the ROS effects of PM2.5 has been shown to alleviate asthma symptoms in a mouse model, suggesting potential translation into clinical practice for PM2.5-induced respiratory complications in patients with asthma [16].

At the molecular level, it is increasingly clear that asthma represents a heterogeneous disease with multiple phenotypes and endotypes [17,18,19]. Nevertheless, it is well known that the disease is mediated by increased bronchial inflammation and hyperresponsiveness, and it is plausible that there is a response to PM pollution with increased systemic inflammation in addition to epithelial airway inflammation. The reasons why exposure to PM in asthmatic patients is linked to a worsening of their condition, expressed as a greater number of exacerbations and visits to emergency departments, and their possible relationship with the OP of certain components present in PM remain to be studied in depth. Interestingly, Canova et al. [20] measured PM10 OP as depletion of ascorbic acid (AA), glutathione and uric acid in synthetic airway fluid. They conducted a bi-directional case-crossover study in patients admitted to hospital for asthma/COPD exacerbations and compared the OP on the admission day with that on 14 days before/after admission. PM10 was collected by using stationary samplers located near the hospital. The analyses included 160 exacerbations in 151 patients. PM10 OP was not associated with asthma/COPD admissions, but the authors highlight the use of stationary samplers instead of PM personal sampling methods as a main limitation, as the latter are more accurate but difficult to incorporate in a case-crossover design.

Interleukin 6 (IL-6) is one of the major studied mediators of inflammation. It is a cytokine produced by different cell types, with immune cells and adipose tissue being the most important. Besides the fact that it is one of the most commonly analyzed inflammatory mediators in respiratory diseases, there is consistent evidence of an association between obesity and higher IL-6 levels; and IL-6 constitutes an important proatherogenic biomarker, and it is one of the systemic inflammation biomarkers most consistently associated with a risk of cardiovascular morbidity and mortality [21,22].

IL-10 is the most important cytokine with anti-inflammatory properties. It is secreted by a variety of cells, and its anti-inflammatory effect seems to be mediated by inhibiting the synthesis of many inflammatory proteins, macrophage activation and the antigen presentation [23]. The so-called “Cytokine storm” with abnormal levels of the inflammatory cytokines IL-6 and IL-10 is well known in COVID-19 disease [24], and the balance of pro- and anti-inflammatory statuses, as determined by the IL-6/IL-10 ratio, has been recently established as a consistent marker of severe SARS-CoV-2 infection [25]. In addition, the IL-6/IL-10 ratio seems to perform better than IL-6 alone as a predictor of the severity of primary open-angle glaucoma [26].

We hypothesized that PM may induce oxidative stress and inflammation due to the oxidative capacity of its components, particularly in patients with asthma. With this rationale, we launched the ASTHMA-FENOP study, which included a group of controls without asthma matched by gender and age with asthmatic patients. Our objective was to determine and compare between these two groups the association between the OP of PM by using personal PM samplers and IL-6 levels as a surrogate of systemic inflammation and the IL-6/IL-10 ratio as a surrogate of the balance of pro- and anti-inflammatory statuses.

## 2. Methods

### 2.1. Study Design

We conducted a cross-sectional study on 44 adult asthmatic patients in collaboration with the Pneumology Service of Hospital Universitario Marqués de Valdecilla (HUMV) and Hospital de Liencres (HL), based on the following inclusion criteria: (1) Diagnosis of asthma according to GINA criteria [27], at least 12 months prior to the baseline visit. (2) Stable treatment with inhaled corticosteroids (ICS) with/without long-acting β adrenoceptor agonists (LABAs), for the past 3 months. (3) No exacerbations in the 4 weeks prior to study inclusion. Previous diagnosis of confirmed COPD and being treated with oral steroids for other reasons than asthma were exclusion criteria. Thirty-seven controls (without asthma) matched with asthmatic patients by gender and age (±5 years old), were recruited as a comparison group.

Volunteers’ residences are shown in Figure S1. Most of them lived in the urban area of Santander, while a second subgroup lived in the Maliaño area (Camargo), near some metallurgical plants, constituting an urban–industrial mixed area. The selection of candidates was based on “a priori” different levels of outdoor PM-bound metals, which are the main drivers of PM-OP. A previous study of the research group at these sites, using PM stationary samplers (see the two red circles in Figure S1), showed higher PM-bound metal levels in the urban-industrial area [28].

### 2.2. Recruitment Scheme and PM Personal Sampling

The recruitment scheme covered 3 consecutive days, with 1–4 patients per week, from November 2022 to February 2024. After signing the informed consent, each volunteer received a personal sampler upon arrival on the first day (visit 1). PM personal samples were collected for 24 h using a two-stage personal modular impactor (SKC PMI coarse) capable of sampling PM2.5 and PM10–2.5 filters separately, connected to a personal pump (SKC Aircheck XR5000, SKC Inc., Valley View Road Eighty Four, PA, USA) that operated at a flow rate of 3 L per minute (l pm). Thirty-seven and 25 mm diameter polytetrafluoroethylene (PTFE) membrane filters were used to collect PM2.5 and PM10–2.5 samples, respectively. The sampler pumps were programmed to sample for 24 h to prevent mishandling and were returned on the second day’s visit. On day 3 (lag1, 25–48 h after returning the personal sampler) FeNO was determined and a blood sample was obtained. The protocol for each volunteer is summarized in Table S1.

### 2.3. Oxidative Potential Analysis

The PM2.5 and PM10–2.5 filters were extracted with 5 mL of a phosphate buffer (PB) solution (0.0075 M Na_2_HPO_4_, 0.0025 M NaH_2_PO_4_) for 24 h at 37 °C and filtered using a syringe cartridge. All samples were stored until OP analysis at 4 °C.

Two OP assays were carried out based on the methodology developed by Expósito et al. [29]: the dithiothreitol (DTT) and AA assays. A microplate reader spectrophotometer (Multiskan Skyhigh microplate spectrophotometer, Thermo Fisher Scientific Inc., Singapore) was used for the OP measurements.

Samples were analyzed in triplicate. The detection limits (D.L.) were calculated by multiplying the standard deviation (SD) of the OP-AA or OP-DTT values of 5 blank filters by the *n* − 1 sample two-tailed Student’s t value at 95% confidence level (2.57). Furthermore, the OP-DTT and OP-AA arithmetic mean of blank filters was subtracted from the depletion rate of each PM sample. The D.L., mean of blank filters, and percentage of samples higher than the D.L. are shown in Table S2.

### 2.4. Cytokine Measurement

Fasting blood samples were collected from all participants on visit 3 (from 8:00–9:00 a.m.) and serum was then separated and stored at −80 °C until assayed. Quantification of cytokine profiles was performed for all samples at the same time utilizing the human IL-6 enzyme-linked immunosorbent assay (ELISA) kit ENZ-KIT178-0001 IL-6 and Human IL-10 ADI-900-036 ELISA kit (Enzo Biochem, Inc., Farmingdale, NY, USA) according to the manufacturer’s protocols. Lowest quantification values for IL-6 and IL-10 cytokines were 0.12 and 1.37 pg/mL, respectively. Samples with concentrations below the D.L. (n = 3 for IL-6 and n = 0 for IL-10) were assigned a value equal to half of the lowest quantification limit and were included in the statistical analysis.

### 2.5. Statistical Analysis

Continuous variables were described as mean and SD and/or median and interquartile ranges (IQR). Statistical differences between groups were compared by using the Student’s *t* test (for equal or different variances, depending on the previous result in the Levene test) in the case of mean comparisons. Normality distribution of variables was studied using the Shapiro–Wilk test. Medians were compared using the Mann–Whitney’s u test. Categorical and discrete variables were expressed as percentages, and comparisons were performed with the Chi-square test, using Yates’ correction or Fisher’s exact test, when appropriate.

IL levels and exposure metrics were dichotomously categorized according to their medians and crude and adjusted odds ratios (aORs) with their 95% confidence intervals (CI) were estimated using unconditional logistic regression models. In these models, the ILs binary results (low and high IL levels) were treated as dependent variables and exposures were treated as independent binary variables (0 = lower values; 1 = higher values). Lastly, OP levels were categorized ordinally (low T1, medium T2, high T3) according to tertiles, calculating adjusted dose–response trends (p trends) in addition to aORs. In a parallel approach, adjusted mean differences (MDs) with their 95%CI were calculated using a linear regression model in which the quantitative IL results were treated as the dependent variable, and each OP exposure as a binary variable (0 = lower values; 1 = higher values).

Age (as a continuous variable), sex, study level (ordinally categorized), body mass index (BMI) and FeNO levels were pre-established as confounders to obtain adjusted ORs and MDs. A stratified analysis based on the asthma and non-asthma statuses was pre-established, along with an additional multivariate model for asthmatic patients. In this additional model, results in the Asthma Control Test (ACT), Test of Adherence to Inhalers (TAI), and asthma severity (according to GINA 2023 guideline steps), were also included as confounders.

The level of statistical significance was set at 0.05 and all tests were two-tailed. We used the SPSS statistical software package 22.0 (SPSS, Inc., Chicago, IL, USA) for statistical analyses.

## 3. Results

### 3.1. Description of the Sample and Distribution of Results for ILs and OP Levels

The characteristics of asthmatic patients are summarized in Table S3. The overall mean age was 52.45 years; [SD = 17.42], with ages ranging from 18 to 80 years. 56.8% were women (n = 25) and the rest men (n = 19, 43.2%). The mean score on the ACT was 22.16 points; [SD = 3.8] with a median of 23, and an IQR between 20 and 25 points. Based on these scores, 81.8% had their asthma controlled (≥20 points). Adherence to inhaled maintenance therapy was good (TAI 10 items = 50) in the majority of patients (n = 33, 75.0%). Most of the sample (n = 25, 56.8%) was in GINA stage 4 (medium dose maintenance of ICS-long-acting β adrenoceptor agonists (LABAs)).

Mean age and sex were similar for both asthmatic patients and controls (mean = 52.45, 56% female) as a result of matching. Most of volunteers were non-smokers (77.3% and 78.4%) being former smokers the rest. University study level was different between asthmatic patients and control volunteers, with a higher prevalence of University studies in controls (*p* < 0.001). Regarding BMI, 36.4% of asthmatic volunteers were on healthy weight according to WHO classification (cut off points 18.5–24.9). Prevalence of overweight (40.9%, BMI 25–29.9) and obesity (22.7%, BMI ≥ 30) was slightly higher among asthmatic volunteers (*p* = 0.096). FeNO median levels (on day 2) were 27 ppb in asthmatic volunteers with a 72.7% having FeNO levels ≥ 20 ppb. As expected, FeNO levels were higher among asthmatic patients compared to controls. See Table S4.

The distribution of IL-6 levels and IL-6/IL-10 ratios, and OP results presented positive asymmetry with the mean values greater than the medians. Median levels for IL-6 and IL-6/IL-10 ratios were slightly higher among controls with statistically significant *p* values. Median levels for PM-OP determinations were higher among asthmatic patients compared to controls, yielding statistical significance in some cases. See Table 1.

### 3.2. Adjusted Associations Between PM-OP, IL-6 and the IL-6/IL-10 Ratio

**Figure 1:**
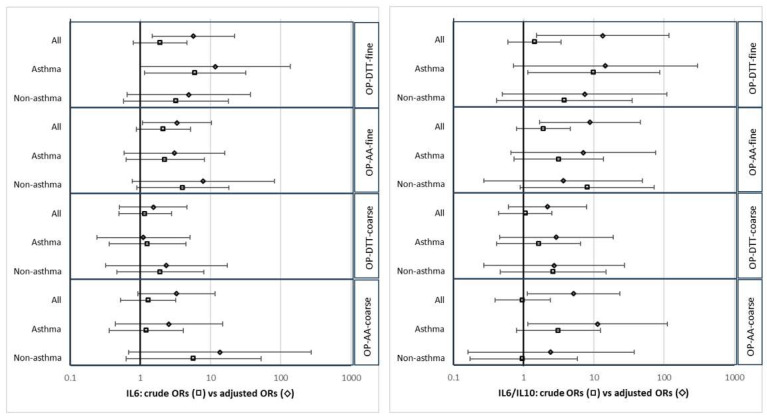
Forest plot of crude and adjusted odds ratios (ORs) between higher values of OP-DTT and OP-AA (for the fine and coarse PM fractions); and higher IL-6 levels (on the **left**) and higher IL-6/IL-10 ratio levels (on the **right**). ORs adjusted for age, sex, educational level, BMI according to WHO classification, and FeNO levels.

**Figure 2:**
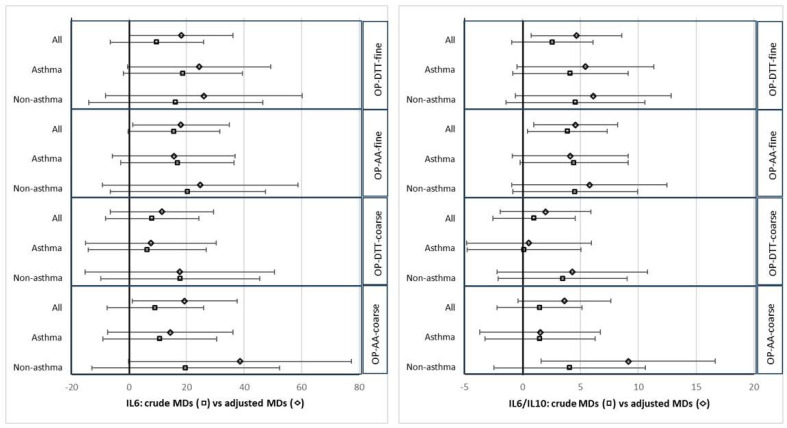
Forest plot of crude and adjusted mean differences (aMDs) between higher values of OP-DTT and OP-AA (for the fine and coarse PM fractions); and IL-6 (on the **left**) and the IL-6/IL-10 ratio levels (on the **right**). MDs adjusted for age, sex, educational level, BMI according to WHO classification, and FeNO levels.

Positive associations for IL-6 levels in the form of adjusted ORs were obtained for all the PM-OP metrics, reaching statistical significance for both OP-DTT and OP-AA in the fine fraction, with differences between crude and adjusted results (associations were higher after adjusting for confusion) (see Tables S5 and S6, and Figure 1). Overall, volunteers with higher OP-DTT values (above median) had a 5.66 fold increased risk of elevated IL-6 levels: adjusted OR = 5.66; 95%CI (1.46 to 21.92); and those with higher OP-AA levels a 3.32 fold increased risk: adjusted OR = 3.32; 95%CI (1.07 to 10.35) (see Table S6 and Figure 1). Associations between OP values and the IL-6/IL-10 ratio were even stronger (see Tables S7 and S8), with statistical significance also reached for OP-AA in the coarse fraction: adjusted OR = 5.14; 95%CI (1.13 to 23.40) (see Table S8 and Figure 1). In the form of adjusted MDs, statistically significant positive MDs were also obtained, indicating higher IL6 and IL-6/IL-10 ratio values among those with higher PM-OP exposures (see Tables S9–S12 and Figure 2). When stratifying the analysis into asthma and controls, positive associations in the form of adjusted ORs and adjusted MDs were found in both groups.

Table 2 presents OR results for IL-6 specifically for the 44 asthmatic patients, examining dose–response patterns by classifying exposure according to tertiles, and after adjusting for specific clinical confounders such as the ACT and TAI scores, or GINA stage. Positive dose–response patterns were obtained for all PM-OP metrics (the greater PM-OP, the greater the association for higher IL-6 levels), with statistically significant adjusted p-trends in the fine fraction for both PM-OP metrics (adjusted p-trend = 0.029 for OP-DTT and 0.01 for OP-AA). Dose–response patterns were maintained for the IL-6/IL-10 ratio (See Table 3).

## 4. Discussion

Among air pollutants, particulate matter (PM) has the greatest impact on human health. We have found an association between the OP of PM and serum IL-6 levels, with positive associations in the form of adjusted MDs and adjusted ORs for all the OP metrics, reaching statistical significance and with well-defined dose–response patterns in the fine fraction. When comparing adults with and without asthma, positive associations were observed in both groups. Lastly, our results were maintained for the IL-6/IL-10 ratio as a surrogate of pro- and anti-inflammatory statuses.

Each PM-OP assay has different sensitivities to the chemical composition of PM. Thus, both OP-DTT and OP-AA are very sensitive to soluble Cu. However, OP-DTT is sensitive to Mn but not to Fe, whereas Fe is an important driver of OP-AA [5]. Regarding the organic species bound to PM, quinones are significant drivers of both OP-DTT and OP-AA, whereas OP-DTT is in general much more sensitive to other organic compounds, particularly photochemically aged organic species. Therefore, PM samples with higher levels of certain transition metals such as Cu, Fe or Mn and some OP-sensitive organic compounds, may result in higher OP values independently of their PM mass concentration. In this way, Weichenthal et al. [30] found that the strength of association between PM2.5 and the risk of acute cardiovascular events was highly influenced by the levels of PM-bound transition metals and S (which is usually correlated with some of these transition metals), because OP metrics were strongly correlated with these metals.

Our results support an association between PM-OP and inflammation as a whole, as we found associations between OP and IL levels for both OP assays (OP-DTT and OP-AA). However, our findings suggest that the OP of the fine fraction plays a more important role in causing systemic inflammation compared to the coarse fraction. This makes sense, considering that the fine fraction can penetrate deeper into the smaller airways. On the other hand, our results, based predominantly on a population living in a mixed urban-industrial area, showed lower PM-OP levels than those reported in other studies [31]. It is conceivable to extrapolate that stronger associations might have been found in a more exposed population.

When we analyzed PM-OP using the stationary samplers located in Maliaño and Santander (the urban-industrial and urban sites, respectively, as shown in Figure S1), we observed differences, with higher levels of PM-OP and metals in the stationary samples from the urban-industrial site compared to those from the urban site. However, for the personal samples assessed in the present study, no spatial pattern was found, suggesting that work and/or leisure activities (hobbies) outside the place of residence substantially contribute to the individual personal exposure. Therefore, since no differences in the geographic distribution of places of residence were observed between asthmatic and non-asthmatic volunteers, the lower levels of PM-OP in non-asthmatic volunteers may be related to different educational levels (higher university studies in non-asthmatics), indicating different occupations or hobbies. Nevertheless, it is difficult to interpret with a single measurement per person, as in our current cross-sectional approach; so, this is a shortcoming that needs to be improved in future prospective studies.

We have presented both crude results and after adjusting for predefined confounders. Table S13 shows the percentage change in the OR after including each predefined confounding variable for the main analyses. We maintained all the predefined variables in the final multivariate model as stated in the research protocol in order to prevent selective reporting bias. The differences observed between the crude (unadjusted) and adjusted MDs and ORs highlight the importance of controlling for confounding bias using multivariate regression models. Because of this, we included asthma-specific clinical variables such as severity of asthma (GINA stage) and the ACT and TAI results in a multivariate regression model restricted to asthmatic patients. We found a clear dose–response pattern for the OP of the fine fraction, with adjusted ORs that reached statistical significance based on both the OP-DTT and OP-AA methods (adjusted p-trends 0.029 and 0.01, respectively). The dose–response pattern is one of Bradford Hill’s classic causality criteria and would therefore support the association between higher OP exposure and elevated IL-6 and IL-6/IL-10 levels. Beyond statistical significance, the existence of a dose–response pattern provides additional support for a real causal association.

Studies evaluating the relationship between PM exposure and blood IL-6 levels have reported mixed results. While most studies [6,21,22,23,32,33,34,35] have found a positive association, others [36,37,38,39,40] have observed no relationship. These contradictory results may be due to the different characteristics of study participants in relation to age, use of healthy volunteers versus volunteers with a disease as inclusion criterion, the study design (observational versus experimental with different compositions and concentrations of PM), the matrix used to determine IL levels (serum, plasma, other matrices…), or the different approaches for controlling confounding bias.

Among these studies, to our knowledge, only three have included asthmatic patients. Urch et al. [34] measured IL-6 blood levels before and after exposures in their experimental study, using a concentrated ambient particle (CAP) facility for PM2.5, in 10 mild asthmatic and 13 non-asthmatic individuals (18–40 years old). They observed an increase in IL-6 blood levels three hours post exposure, but only after CAP alone exposures (without ozone), and the IL-6 increase was associated with increased PM2.5 mass concentration, suggesting a dose–response pattern. The responses of asthmatic and non-asthmatic volunteers were similar. In contrast, Brown et al. [38] found no association between asthmatic children (aged 6–17 years old) living near a major roadway and IL-6 or IL-10 plasma levels. Lastly, Klümper et al. [23] recruited 27 children with asthma and 59 without asthma (all aged 6-year-old). Their findings showed a differential response between the two groups. No association was found between air pollution and IL-6 or IL-10 in non-asthmatic children. However, the mean ratios for asthmatic children tended to be greater than 1, suggesting that children with asthma are more susceptible to traffic-related air pollution exposure, compared to non-asthmatic children.

An important methodological strength of our study is the use of PM personal samplers, also known as personal environmental monitors (PEM), to characterize the individual PM-OP exposure in each volunteer. It consists of a portable impactor connected to a personal pump. The impactor enabled the separation of fine (PM2.5) and coarse (PM10–2.5) particles and for each particle size the two OP assays (DTT and AA) were performed. As mentioned in the introduction section, there are very few studies that have characterized PM-OP using personal samplers [8,9,10,11,41]. To our knowledge, only two studies have utilized PM personal samplers specifically in asthmatic patients. Both studies focused on children, and neither measured PM-OP. In the study by Isiugo et al. [42], the children did not carry the personal samplers. Instead, the personal samplers were placed 48 h outdoors and in the bedrooms of the children’s homes (indoors), and only spirometries were performed to determine lung function. In the study published by Delfino et al. [43], 45 children carried personal samplers, but only FeNO was determined. Therefore, our study is the first one on the association between the PM-OP obtained using personal samplers and ILs blood levels. In contrast, an important limitation of our study is the sample size and the statistical power to detect positive associations as statistically significant. Because of this, most of the statistically significant associations observed in the total sample (n = 81), lost statistical significance in the stratified analysis (n = 44 asthmatics and n = 37 controls) with wider 95% CI.

For clinical management, asthmatic patients are classified into type 2-high and type 2-low. In type 2-high asthma, type 2 inflammation is present with its associated cytokines IL-4, IL-5, and IL-13, high levels of blood and/or sputum eosinophils, and elevated FeNO. Type 2-low asthma is defined by the absence of type 2 markers. Both types of inflammation are not mutually exclusive and overlapping phenotypes can occur in asthmatic patients [17,18,19]. Genome-wide association studies and other basic research have shown an association between IL-6 signaling and asthma [44,45], which is also supported by clinical studies [46]; and there is growing evidence of subgroups of type 2-low asthma where IL-6 plays a major role. These patients might benefit from another biologic targeting IL-6 signaling such as Olamkicept [47]. Our ASTHMA-FENOP study was designed to compare results between asthmatic and non-asthmatic adults, based on the rationale that inflammation in response to PM-OP may differ between these two groups. IL-6 levels (as well as IL-6/IL-10 ratio) were slightly higher in our non-asthmatic compared to asthmatic patients, with medians of 9.24 and 7.86 pg/mL, respectively. However, no big differences were found when comparing the associations between PM-OP and IL-6 levels or the IL-6/IL-10 ratio. This suggests that higher PM-OP increases IL-6 levels and the IL-6/IL-10 ratio in both asthmatic and non-asthmatic adults.

## 5. Conclusions

We found an independent association between PM-OP and systemic inflammation, as determined by IL-6 levels and the IL-6/IL-10 ratio, in both asthmatic and non-asthmatic volunteers after adjusting for confounding variables; and with a dose-response pattern that suggests causality and supports that asthma is a heterogeneous disease at the molecular level. Future studies with a larger number of subjects will be needed to clarify the association between higher PM-OP exposure and levels of IL-6 and IL-6/IL-10 ratio, as well as their potential clinical implications.

## Figures and Tables

**Table 1:** Description of systemic inflammation based on ILs levels and PM-OP metrics as a function of their asthma or control statuses.

	Asthma		Non-Asthma		All		
	N = 44		N = 37		N = 81		*p Value*
**Systemic inflammation**							
IL-6 pg/mL. Mean [SD]	18.08	32.57	30.33	40.45	23.68	36.66	*0.135*
IL-6 pg/mL. Median [IQR]	5.84	2.06–15.25	10.56	7.86–38.9	9.24	4.08–16.70	*0.009*
IL-10 pg/mL. Mean [SD]	10.95	19.22	5.60	4.15	8.51	14.61	*0.079*
IL-10 pg/mL. Median [IQR]	6.35	3.73–10.18	4.48	3.80–5.32	4.74	3.73–7.40	*0.041*
IL-6/IL-10 ratio. Mean [SD]	3.57	7.77	6.05	8.15	4.70	7.99	*0.166*
IL-6/IL-10 ratio. Median [IQR]	0.95	0.26–2.03	2.44	1.52–8.00	1.52	0.66–3.27	*<0.001*
**PM-OP metrics (nmol/min/m^3^)**							
OP-DTT PM2.5. Mean [SD]	0.30	0.29	0.17	0.25	0.24	0.27	*0.029*
OP-DTT PM2.5. Median [IQR]	0.24	0.15–0.34	0.10	0.03–0.18	0.16	0.1–0.31	*<0.001*
OP-AA PM2.5. Mean [SD]	0.72	1.29	0.34	0.89	0.55	1.14	*0.127*
OP-AA PM2.5. Median [IQR]	0.23	0.12–0.49	0.15	0.06–0.28	0.18	0.07–0.37	*0.027*
OP-DTT PM10–2.5. Mean [SD]	0.18	0.11	0.14	0.11	0.16	0.11	*0.058*
OP-DTT PM10–2.5. Median [IQR]	0.17	0.10–0.26	0.11	0.06–0.19	0.13	0.08–0.22	*0.052*
OP-AA PM10–2.5. Mean [SD]	0.59	1.38	0.17	0.11	0.40	1.04	*0.051*
OP-AA PM10–2.5. Median [IQR]	0.22	0.10–0.55	0.20	0.1–0.20	0.20	0.1–0.39	*0.029*

SD = standard deviation. IQR = interquartile rank.

**Table 2:** Association between PM-OP metrics and IL-6 levels, restricted to asthmatic patients and studying dose–response patterns.

		IL-6 pg/mL (Median)									
		n = 27	n = 17								
*PM-OP_v_* nmol min^−1^ m^−3^	*Cut-Off Point*	≤9.24	9.24+	OR Crude	95%	CI	*p Value*	aOR	95%	CI	*p Value*
***OP-DTT PM2.5***											
Lower values	≤0.161	12	2	1				1			
Higher values	0.161+	15	15	6.00	1.14	31.53	*0.034*	39.70	2.19	718.60	*0.013*
***OP-DTT PM2.5 (Tertiles)***											
Low values	≤0.110	4	1	1				1			
Medium values	0.111–0.236	11	7	2.55	0.23	27.71	*0.443*	4.52	0.25	83.26	*0.310*
High values	0.236+	12	9	3.00	0.29	31.63	*0.361*	41.96	1.12	1566.43	*0.043*
*Linear p-trend*							*0.409*				*0.029*
***OP-AA PM2.5***											
Lower values	≤0.184	13	5	1				1			
Higher values	0.184+	14	12	2.23	0.62	8.08	*0.223*	3.76	0.75	18.92	*0.108*
***OP-AA PM2.5 (Tertiles)***											
Low values	≤0.125	8	3	1				1			
Medium values	0.126–0.260	13	3	0.62	0.10	3.82	*0.602*	0.57	0.06	5.62	*0.632*
High values	0.260+	6	11	4.89	0.93	25.67	*0.061*	48.39	2.12	1106.24	*0.015*
*Linear p-trend*							*0.032*				*0.01*
***OP-DTT PM10–2.5***											
Lower values	≤0.129	11	6	1				1			
Higher values	0.129+	16	11	1.26	0.36	4.43	*0.718*	1.82	0.36	9.26	*0.469*
***OP-DTT PM10–2.5 (Tertiles)***											
Low values	≤0.090	6	4	1				1			
Medium values	0.091–0.200	9	6	1.00	0.20	5.12	*1.000*	1.33	0.20	9.02	*0.769*
High values	0.200+	12	7	0.88	0.18	4.21	*0.868*	1.46	0.20	10.90	*0.709*
*Linear p-trend*							*0.851*				*0.713*
***OP-AA PM10–2.5***											
Lower values	≤0.200	14	8	1				1			
Higher values	0.200+	13	9	1.21	0.36	4.08	*0.757*	5.16	0.78	34.15	*0.089*
***OP-AA PM10–2.5 (Tertiles)***											
Low values	≤0.100	7	4	1				1			
Medium values	0.101–0.300	8	5	1.09	0.21	5.76	*0.916*	0.88	0.11	6.85	*0.905*
High values	0.300+	12	8	1.17	0.26	5.33	*0.842*	4.53	0.47	43.47	*0.191*
*Linear p-trend*							*0.842*				*0.221*

OR = odds ratio. aOR = OR adjusted for age, sex, ACT, TAI, severity of asthma, FeNO levels and BMI according to WHO classification.

**Table 3:** Association between PM-OP metrics and the IL-6/IL-10 ratio, restricted to asthmatic patients and studying dose–response patterns.

		IL-6/IL-10 (Median)									
		n = 30	n = 14								
*PM-OP_v_* nmol min^−1^ m^−3^	*Cut-Off Point*	≤1.52	1.52+	OR Crude	95%	CI	*p Value*	aOR	95%	CI	*p Value*
***OP-DTT PM2.5***											
Lower values	≤0.161	13	1	1				1			
Higher values	0.161+	17	13	9.94	1.15	86.06	*0.037*	14.65	1.15	186.87	*0.039*
***OP-DTT PM2.5 (Tertiles)***											
Low values	≤0.110	5	1	1				1			
Medium values	0.111–0.236	14	3	1.07	0.09	12.83	*0.957*	0.51	0.02	10.52	*0.660*
High values	0.236+	11	10	4.55	0.45	45.86	*0.199*	9.39	0.44	198.71	*0.150*
*Linear p-trend*							*0.063*				*0.074*
***OP-AA PM2.5***											
Lower values	≤0.184	15	3	1				1			
Higher values	0.184+	15	11	3.67	0.85	15.84	*0.082*	5.20	0.78	34.54	*0.088*
***OP-AA PM2.5 (Tertiles)***											
Low values	≤0.125	10	1	1				1			
Medium values	0.126–0.260	12	4	2.31	0.21	25.66	*0.496*	6.66	0.25	177.74	*0.258*
High values	0.260+	8	9	11.25	1.17	108.41	*0.036*	33.86	1.08	1066.42	*0.045*
*Linear p-trend*							*0.015*				*0.018*
***OP-DTT PM10–2.5***											
Lower values	≤0.129	12	5	1				1			
Higher values	0.129+	18	9	1.20	0.32	4.47	*0.786*	1.30	0.26	6.63	*0.75*
***OP-DTT PM10–2.5 (Tertiles)***											
Low values	≤0.090	7	3	1				1			
Medium values	0.091–0.200	11	4	0.85	0.14	4.99	*0.856*	0.59	0.08	4.36	*0.607*
High values	0.200+	12	7	1.36	0.26	7.04	*0.713*	1.18	0.17	8.07	*0.867*
*Linear p-trend*							*0.640*				*0.814*
***OP-AA PM10–2.5***											
Lower values	≤0.200	17	5	1				1			
Higher values	0.200+	13	9	2.35	0.64	8.73	*0.200*	4.69	0.84	26.26	*0.079*
***OP-AA PM10–2.5 (Tertiles)***											
Low values	≤0.100	7	4	1				1			
Medium values	0.101–0.300	11	2	0.32	0.05	2.22	*0.248*	0.25	0.03	2.41	*0.232*
High values	0.300+	12	8	1.17	0.26	5.33	*0.842*	2.10	0.28	15.51	*0.467*
*Linear p-trend*							*0.652*				*0.430*

OR = odds ratio. aOR = OR adjusted for age, sex, ACT, TAI, severity of asthma, FeNO levels and BMI according to WHO classification.

## Data Availability

Data cannot be made publicly available in order to protect patient privacy. The data are available on request from the University of Cantabria Archive (http://repositorio.unican.es/, accessed on 25 November 2024) for researchers who meet the criteria for access to confidential data. Requests may be sent to the Ethics Committee (ceicc@idival.org), or Miguel Santibañez (santibanezm@unican.es).

## Supplementary Materials

The following supporting information can be downloaded at: https://www.mdpi.com/article/10.3390/antiox13121464/s1, Figure S1: Location of volunteers’ residences (blue points) and the two stationary sampling points (urban and urban-industrial) (red points) established by the research group; Table S1: Visit protocol for the volunteers (n = 81); Table S2: PM-OP detection limits (D.L), mean of blank filters, and percentage of samples higher than the D.L.; Table S3: Description of asthmatic patients as a function of gender.; Table S4: Description of the sample as a function of their asthma or control statuses.; Table S5: Crude association between PM-OP metrics and IL-6 levels, overall and as a function of their asthma or control statuses; Table S6: Adjusted association between PM-OP metrics and IL-6 levels, overall and as a function of their asthma or control statuses.; Table S7: Crude association between PM-OP metrics and the IL-6/IL-10 ratio, overall and as a function of their asthma or control statuses.; Table S8: Adjusted association between PM-OP metrics and the IL-6/IL-10 ratio, overall and as a function of their asthma or control statuses.; Table S9: Crude mean differences (MD) for IL-6 levels, between higher and lower PM-OP values.; Table S10: Adjusted mean differences (aMD) for the IL-6/IL-10 ratio, between higher and lower PM-OP values.; Table S11: Crude mean differences (MD) for IL-6 levels, between higher and lower PM-OP values.; Table S12: Adjusted mean differences (aMD) for the IL-6/IL-10 ratio, between higher and lower PM-OP values.; Table S13: % of change in the OR for high IL-6 levels, after including each predefined confounding variable, in all patients (n = 81).
