# Supplementary material for: Association Between Oxidative Potential of Particulate Matter Collected by Personal Samplers and Systemic Inflammation Among Asthmatic and Non-Asthmatic Adults
Source: Antioxidants (Basel). 2024 Nov 28;13(12):1464. doi: 10.3390/antiox13121464 (PMC11673029; doi:10.3390/antiox13121464)
Supplement: Supplementary file 1 [file antioxidants-13-01464-s001.zip › antioxidants-3284958-supplementary.pdf]

Supplementary Materials

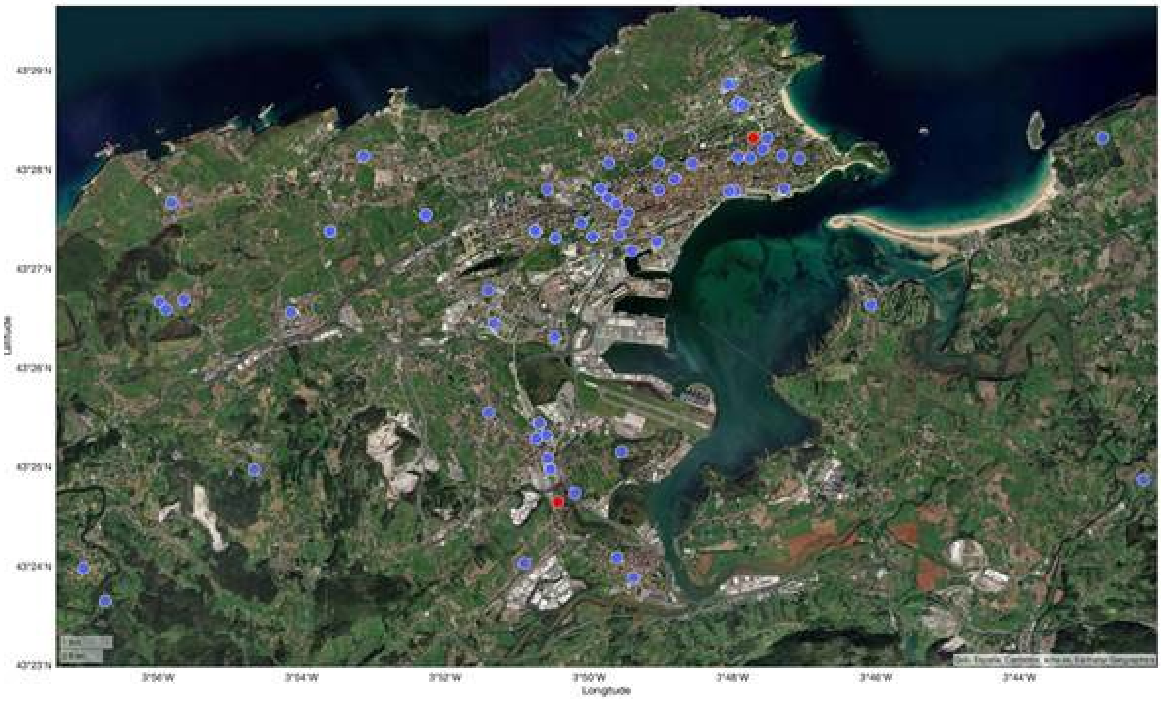

Figure S1. Location of volunteers' residences (blue points) and the two stationary sampling points (urban and urban-industrial) (red points) established by the research group.

Table S1. Visit protocol for the volunteers (n=81)

**Day 1. Visit 1.**	**Day 2 (lag0, after 24 hours of PM personal monitoring). Visit 2.**	**Day3 (lag1, after 25-48 hours). Visit 3.**
Informed consent form<br>Questionnaire<br>PM personal monitor delivery	Return of the PM personal monitor<br>Questionnaire (summary of activities done with the PM personal monitor)	Blood collection 8:00-9:00h (IL-6 and IL-10)<br>FeNO

Table S2. PM-OP detection limits (D.L), mean of blank filters, and percentage of samples higher than the D.L.

	Blank mean (μM/min)		D.L. (μM/min)		% of n>D.L.	
	**Asthma**	**Control**	**Asthma**	**Control**	**Asthma**	**Control**
OP-DTT PM2.5	0.10	0.11	0.049	0.042	92	68
OP-AA PM2.5	0.23	0.12	0.081	0.034	73	78
OP-DTT PM 10-2.5	0.10	0.12	0.036	0.036	86	78
OP-AA PM10-2.5	0.21	0.11	0.051	0.022	73	89

Table S3. Description of asthmatic patients as a function of gender.

	Women Asthma N=25		Men Asthma N=19		All Asthma N=44		*p value*
Age, yrs. Mean [SD]	51.68	17.76	53.47	17.40	52.45	17.42	*0.739*
Age, yrs. Median [IQR]	50	38-69	50	40-70	50	40-69	*0.713*
FEV1%. Mean [SD]	89.04	16.883	97.21	17.018	92.57	17.24	*0.122*
FEV1%. Median [IQR]	91	77-105.5	92	87-106	91	82-105.8	
ACT. Mean [SD]	22.04	4.24	22.32	3.23	22.16	3.8	*0.815*
ACT. Median [IQR]	23	20.5-25	23	20-25	23	20.3-25	*0.98*
ACT≤19 Uncontrolled asthma	5	20.0%	3	15.8%	8	18.2%	*0.72*
ACT≥20 Controlled asthma	20	80.0%	16	84.2%	36	81.8%	
Number of moderate-severe exacerbations without hospital admission (previous 12 months)							
0	17	68.0%	17	89.5%	34	77.3%	*0.107*
1	4	16.0%	1	5.3%	5	11.4%	
2	4	16.0%	0		4	9.1%	
4	0		1	5.3%	1	2.3%	
Number of severe exacerbations with hospital admission (previous 12 months)							
0	23	92.0%	18	94.7%	41	93.2%	*0.668*
1	1	4.0%	1	5.3%	2	4.5%	
2	1	4.0%	0		1	2.3%	
Number of severe exacerbations with ICU admission (previous 12 months)							
0	25	100.0%	19	100.0%	44	100.0%	
Need for systemic corticosteroids							
No	17	68.0%	16	84.2%	33	75.0%	*0.219*
Yes	8	32.0%	3	15.8%	11	25.0%	
TAI 10 Items. Mean [SD]	49.64	0.81	49.21	1.55	49.45	1.19	*0.24*
TAI 10 Items. Median [IQR]	50	50-50	50	49-50	50	49.25-50	*0.349*
Low adherence (≤49 points)	5	20.0%	6	31.6%	11	25.0%	*0.38*
Poor adherence (≤45 points)	0		1	5.3%	1	2.3%	*0.428*
Intermediate adherence (46-49 points)	5	20.0%	5	26.3%	10	22.7%	
Good adherence (50 points)	20	80.0%	13	68.4%	33	75.0%	
GINA stage							
3	4	16.0%	1	5.3%	5	11.4%	*0.514*
4	13	52.0%	12	63.2%	25	56.8%	
5	8	32.0%	6	31.6%	14	31.8%	
GEMA stage							
3	4	16.0%	1	5.30%	5	11.4%	*0.641*
4	10	40.0%	8	42.1%	18	40.9%	
5	7	28.0%	5	26.3%	12	27.3%	
6	4	16.0%	5	26.3%	9	20.5%	
Biologic treatment							
No	21	84.0%	14	73.7%	35	79.5%	*0.401*
Yes	4	16.0%	5	26.3%	9	20.5%	
FeNO≥20ppb							
No	8	32.0%	4	21.1%	12	27.3%	*0.419*
Yes	17	68.0%	15	78.9%	32	72.7%	
Blood Eosinophils ≥150 cells/mm^3							
No	6	24.0%	6	31.6%	12	27.3%	*0.576*

Yes	19	76.0%	13	68.4%	32	72.7%	
Blood Neutrophils ≥5000 cells/mm^3							
No	22	88.0%	15	78.9%	37	84.1%	*0.416*
Yes	3	12.0%	4	21.1%	7	15.9%	
Prick test							
Negative	16	64.0%	11	57.9%	27	61.4%	*0.68*
Positive at least in one antigen	9	36.0%	8	42.1%	17	38.6%	
IgE ≥250 IU/ml							
No	20	80.0%	10	52.6%	30	68.2%	*0.054*
Yes	5	20.0%	9	47.4%	14	31.8%	

SD = standard deviation. IQR = interquartile rank. ACT= Asthma control test. TAI= Test of adherence to inhalers. GINA= Global initiative for Asthma. Global Strategy for Asthma Management and Prevention, 2023. GEMA= Spanish Guideline on the Management of Asthma v5.3.

Table S4. Description of the sample as a function of their asthma or control status.

	**Asthma**		**Non-asthma**		**All**		
	**N=44**		**N=37**		**N=81**		***p value***
Age, yrs. Mean [SD]	52.45	17.42	52.03	16.69	52.26	16.99	*0.911*
Age, yrs. Median [IQR]	50	40-69	54	39-67	52	39.5-68.5	*0.794*
No smoker	34	77.3%	29	78.4%	63	77.8%	*0.905*
Former smoker	10	22.7%	8	21.6%	18	22.2%	
Sex at birth							
Female	25	56.80%	21	56.8%	46	56.8%	*1*
Male	19	43.20%	16	43.2%	35	43.2%	
Study level							
Primary education	5	11.4%	1	2.7%	6	7.4%	*<0.001*
Secondary education	13	29.5%	3	8.1%	16	19.8%	
High school level	16	36.4%	2	5.4%	18	22.2%	
University studies	10	22.7%	31	83.8%	41	50.6%	
BMI (WHO classification)							
Healthy Weight 18.5-24.9	16	36.4%	21	56.8%	37	45.7%	*0.096*
Overweight 25-29.9	18	40.9%	13	35.1%	31	38.3%	
Obesity ≥30	10	22.7%	3	8.1%	13	16.0%	
FeNO, ppb. Mean [SD]	37.27	24.23	24.43	14.49	31.41	21.25	*0.004*
FeNO, ppb. Median [IQR]	27	19-52	21	15-29.5	23	16-40.5	*0.013*
FeNO≥20ppb							
No	12	27.3%	17	*45.9%*	29	*35.8%*	*0.081*
Yes	32	72.7%	20	*54.1%*	52	*64.2%*	
Blood Eosinophils ≥150 cells/mm^3							
No	12	27.3%	13	35.1%	25	30.9%	*0.445*
Yes	32	72.7%	24	64.9%	56	69.1%	
Blood Neutrophils ≥5000 cells/mm^3							
No	37	84.1%	35	94.6%	72	88.9%	*0.134*
Yes	7	15.9%	2	5.4%	9	11.1%	

SD = standard deviation. IQR = interquartile rank.

Table S5. Crude association between PM-OP metrics and IL-6 levels, overall and as a function of their asthma or control status.

		IL-6 pg/mL (Median)		All (n=81)				Asthma (n=44)				Non-asthma (controls) (n=37)			
		n=41	n=40												
PM-OP$_v$ nmol min^{-1} m^{-3}	*Cut-off point*	≤ 9.24	9.24+	OR	95%	CI	*p value*	OR	95%	CI	*p value*	OR	95%	CI	*p value*
OP-DTT PM2.5															
Lower values	≤ .161	24	17	1				1				1			
Higher values	.161+	17	23	1.91	0.79	4.62	*0.151*	6.00	1.14	31.53	*0.051*	3.20	0.57	17.97	*0.186*
OP-AA PM2.5															
Lower values	≤ .184	24	16	1				1				1			
Higher values	.184+	17	24	2.12	0.87	5.14	*0.097*	2.23	0.62	8.08	*0.188*	4.00	0.88	18.22	*0.073*
OP-DTT PM10-2.5															
Lower values	≤ .129	21	19	1				1				1			
Higher values	.129+	20	21	1.16	0.49	2.78	*0.738*	1.26	0.36	4.43	*0.909*	1.92	0.46	7.98	*0.368*
OP-AA PM10-2.5															
Lower values	≤ .200	27	24	1				1				1			
Higher values	.200+	14	16	1.29	0.52	3.17	*0.586*	1.21	0.36	4.08	*0.299*	5.69	0.62	52.34	*0.125*

OR=Crude Odds Ratio

Table S6. Adjusted association between PM-OP metrics and IL-6 levels, overall and as a function of their asthma or control status.

		**IL-6 pg/mL (Median)**		**All (n=81)**				**Asthma (n=44)**				**Non-asthma (controls) (n=37)**			
		n=41	n=40												
PM-OP$_v$ nmol min^{-1} m^{-3}	*Cut-off point*	≤ 9.24	9.24+	aOR	95%	CI	*p value*	aOR	95%	CI	*p value*	aOR	95%	CI	*p value*
OP-DTT PM2.5															
Lower values	≤ .161	24	17	1				1				1			
Higher values	.161+	17	23	5.66	1.46	21.91	*0.012*	11.62	0.98	137.25	*0.051*	4.90	0.65	37.12	*0.124*
OP-AA PM2.5															
Lower values	≤ .184	24	16	1				1				1			
Higher values	.184+	17	24	3.32	1.07	10.35	*0.038*	3.05	0.58	16.00	*0.188*	7.80	0.76	80.56	*0.085*
OP-DTT PM10-2.5															
Lower values	≤ .129	21	19	1				1				1			
Higher values	.129+	20	21	1.52	0.50	4.59	*0.456*	1.09	0.24	5.07	*0.909*	2.34	0.32	17.30	*0.406*
OP-AA PM10-2.5															
Lower values	≤ .200	27	24	1				1				1			
Higher values	.200+	14	16	3.25	0.92	11.54	*0.068*	2.54	0.44	14.79	*0.299*	13.60	0.68	271.98	*0.088*

aOR= Odds Ratio adjusted for asthma/control status, age, sex, study level, BMI according to WHO classification and FeNO levels

Table S7. Crude association between PM-OP metrics and the IL-6/IL-10 ratio, overall and as a function of their asthma or control status.

		**IL-6/IL10 (Median)**		**All (n=81)**				**Asthma (n=44)**				**Non-asthma (controls) (n=37)**			
		n=40	n=41												
PM-OP$_v$ nmol min^{-1} m^{-3}	*Cut-off point*	≤ 1.52	1.52+	OR	95%	CI	*p value*	OR	95%	CI	*p value*	OR	95%	CI	*p value*
OP-DTT PM2.5															
Lower values	≤ .161	22	19	1				1				1			
Higher values	.161+	18	22	1.42	0.59	3.39	*0.436*	9.94	1.15	86.06	*0.037*	3.79	0.41	35.07	*0.148*
OP-AA PM2.5															
Lower values	≤ .184	23	17	1				1				1			
Higher values	.184+	17	24	1.91	0.79	4.62	*0.151*	3.13	0.72	13.59	*0.129*	8.00	0.88	72.70	*0.065*
OP-DTT PM10-2.5															
Lower values	≤ .129	20	20	1				1				1			
Higher values	.129+	20	21	1.05	0.44	2.51	*0.913*	1.63	0.41	6.44	*0.490*	2.63	0.46	14.97	*0.277*
OP-AA PM10-2.5															
Lower values	≤ .200	25	26	1				1				1			
Higher values	.200+	15	15	0.96	0.39	2.37	*0.932*	3.12	0.79	12.35	*0.106*	0.96	0.17	5.85	*0.960*

OR=Crude Odds Ratio

Table S8. Adjusted association between PM-OP metrics and the IL-6/IL-10 ratio, overall and as a function of their asthma or control status.

		**IL-6/IL10 (Median)**		**All (n=81)**				**Asthma (n=44)**				**Non-asthma (controls) (n=37)**			
		n=40	n=41												
PM-OP$_v$ nmol min^{-1} m^{-3}	*Cut-off point*	≤ 1.52	1.52+	aOR	95%	CI	*p value*	aOR	95%	CI	*p value*	aOR	95%	CI	*p value*
OP-DTT PM2.5															
Lower values	≤ .161	22	19	1				1				1			
Higher values	.161+	18	22	13.41	1.52	118.24	*0.019*	14.62	0.71	302.26	*0.083*	7.38	0.49	110.48	*0.148*
OP-AA PM2.5															
Lower values	≤ .184	23	17	1				1				1			
Higher values	.184+	17	24	8.77	1.68	45.73	*0.01*	7.07	0.66	75.82	*0.106*	3.64	0.27	48.92	*0.33*
OP-DTT PM10-2.5															
Lower values	≤ .129	20	20	1				1				1			
Higher values	.129+	20	21	2.17	0.60	7.83	*0.235*	2.90	0.45	18.69	*0.262*	2.72	0.27	27.12	*0.394*
OP-AA PM10-2.5															
Lower values	≤ .200	25	26	1				1				1			
Higher values	.200+	15	15	5.14	1.13	23.40	*0.034*	11.23	1.14	111.05	*0.039*	2.41	0.16	37.22	*0.529*

aOR= Odds Ratio adjusted for asthma/control status, age, sex, study level, BMI according to WHO classification and FeNO levels

Table S9. Crude mean differences (MD) for IL-6 levels, between higher and lower PM-OP values.

	**IL-6 pg/mL**												
		**All (n=81)**				**Asthma (n=44)**				**Non-Asthma (controls) (n=37)**			
PM-OP$_v$ nmol min^{-1} m^{-3}	*Cut-off point*	MD	95%	CI	*p value*	MD	95%	CI	*p value*	MD	95%	CI	*p value*
OP-DTT PM2.5													
Lower values	≤ .161	0				0				0			
Higher values	.161+	9.71	-6.47	25.88	*0.236*	18.77	-1.95	39.49	*0.075*	16.27	-14.06	46.60	*0.284*
OP-AA PM2.5													
Lower values	≤.184	0				0				0			
Higher values	.184+	15.63	-0.32	31.57	*0.055*	16.88	-2.82	36.59	*0.091*	20.46	-6.53	47.45	*0.133*
OP-DTT PM10-2.5													
Lower values	≤ .129	0				0				0			
Higher values	.129+	8.07	-8.15	24.29	*0.325*	6.34	-14.16	26.83	*0.536*	17.85	-9.72	45.41	*0.197*
OP-AA PM10-2.5													
Lower values	≤ .200	0				0				0			
Higher values	.200+	9.12	-7.65	25.90	*0.282*	10.77	-9.00	30.54	*0.278*	19.73	-12.84	52.29	*0.227*

Table S10. Adjusted mean differences (aMD) for IL-6 levels, between higher and lower PM-OP values.

	**IL-6 pg/mL**												
		**All (n=81)**				**Asthma (n=44)**				**Non-Asthma (controls) (n=37)**			
PM-OP$_v$ nmol min^{-1} m^{-3}	*Cut-off point*	aMD	95%	CI	*p value*	aMD	95%	CI	*p value*	aMD	95%	CI	*p value*
OP-DTT PM2.5													
Lower values	≤ .161	0				0				0			
Higher values	.161+	18.16	0.10	36.22	*0.049*	24.39	-0.44	49.21	*0.054*	26.06	-8.16	60.28	*0.130*
OP-AA PM2.5													
Lower values	≤.184	0				0				0			
Higher values	.184+	18.09	1.32	34.85	*0.035*	15.58	-5.77	36.93	*0.148*	24.68	-9.33	58.69	*0.149*
OP-DTT PM10-2.5													
Lower values	≤ .129	0				0				0			
Higher values	.129+	11.44	-6.47	29.35	*0.207*	7.64	-15.00	30.27	*0.498*	17.65	-15.31	50.60	*0.283*
OP-AA PM10-2.5													
Lower values	≤ .200	0				0				0			
Higher values	.200+	19.32	1.09	37.56	*0.038*	14.36	-7.36	36.08	*0.189*	38.61	-0.14	77.36	*0.051*

MDs adjusted for asthma/control status, age, sex, study level, BMI according to WHO classification and FeNO levels.

Table S11. Crude mean differences (MD) for the IL-6/ IL-10 ratio, between higher and lower PM-OP values.

	**IL-6/IL-10**												
		**All (n=81)**				**Asthma (n=44)**				**Non-Asthma (controls) (n=37)**			
PM-OP$_v$ nmol min^{-1} m^{-3}	*Cut-off point*	MD	95%	CI	*p value*	MD	95%	CI	*p value*	MD	95%	CI	*p value*
OP-DTT PM2.5													
Lower values	≤ .161	0				0				0			
Higher values	.161+	2.59	-0.92	6.10	*0.145*	4.15	-0.82	9.12	*0.099*	4.58	-1.44	10.59	*0.131*
OP-AA PM2.5													
Lower values	≤.184	0				0				0			
Higher values	.184+	3.89	0.44	7.34	*0.028*	4.46	-0.20	9.12	*0.060*	4.55	-0.85	9.95	*0.096*
OP-DTT PM10-2.5													
Lower values	≤ .129	0				0				0			
Higher values	.129+	0.99	-2.57	4.54	*0.582*	0.13	-4.78	5.04	*0.957*	3.48	-2.08	9.05	*0.212*
OP-AA PM10-2.5													
Lower values	≤ .200	0				0				0			
Higher values	.200+	1.50	-2.17	5.16	*0.420*	1.52	-3.24	6.28	*0.523*	4.10	-2.46	10.65	*0.213*

Table S12. Adjusted mean differences (aMD) for the IL-6/ IL-10 ratio, between higher and lower PM-OP values.

	**IL-6/IL-10**												
		**All (n=81)**				**Asthma (n=44)**				**Non-Asthma (controls) (n=37)**			
PM-OP$_v$ nmol min^{-1} m^{-3}	*Cut-off point*	aMD	95%	CI	*p value*	aMD	95%	CI	*p value*	aMD	95%	CI	*p value*
OP-DTT PM2.5													
Lower values	≤ .161	0				0				0			
Higher values	.161+	4.67	0.77	8.57	*0.02*	5.44	-0.46	11.33	*0.070*	6.13	-0.59	12.86	*0.072*
OP-AA PM2.5													
Lower values	≤.184	0				0				0			
Higher values	.184+	4.60	0.99	8.22	*0.013*	4.11	-0.89	9.12	*0.104*	5.79	-0.91	12.49	*0.088*
OP-DTT PM10-2.5													
Lower values	≤ .129	0				0				0			
Higher values	.129+	1.99	-1.93	5.92	*0.315*	0.57	-4.81	5.94	*0.832*	4.32	-2.20	10.83	*0.186*
OP-AA PM10-2.5													
Lower values	≤ .200	0				0				0			
Higher values	.200+	3.61	-0.40	7.62	*0.077*	1.53	-3.70	6.75	*0.558*	9.14	1.61	16.66	*0.019*

MDs adjusted for asthma/control status, age, sex, study level, BMI according to WHO classification and FeNO levels.

Table S13. % of change in the OR for high IL-6 levels, after including each predefined confounding variable, in all patients (n=81)

PM-OP$_v$ nmol min^{-1} m^{-3}	*Cut-off point*	crude OR	aOR (age)	*% change in OR*	aOR (sex)	*% change in OR*	aOR (study level)	*% change in OR*	aOR (BMI)	*% change in OR*	aOR (FeNO levels)	*% change in OR*
OP-DTT PM2.5												
Lower values	≤ .161	1	1		1		1		1		1	
Higher values	.161+	1.91	1.88	*1.43%*	2.16	*-11.61%*	1.94	*-1.65%*	2.02	*-5.35%*	1.90	*0.47%*
OP-AA PM2.5												
Lower values	≤ .184	1	1		1		1		1		1	
Higher values	.184+	2.12	2.08	*1.88%*	2.09	*1.58%*	2.33	*-9.06%*	2.10	*0.81%*	2.15	*-1.53%*
OP-DTT PM10-2.5												
Lower values	≤ .129	1	1		1		1		1		1	
Higher values	.129+	1.16	1.17	*-0.51%*	1.25	*-7.05%*	1.26	*-8.08%*	1.12	*3.94%*	1.17	*-0.51%*
OP-AA PM10-2.5												
Lower values	≤ .200	1	1		1		1		1		1	
Higher values	.200+	1.29	1.41	*-8.92%*	1.41	*-8.60%*	1.44	*-10.94%*	1.20	*7.62%*	1.27	*1.26%*

aOR= Odds Ratio adjusted for the indicated variable
